# Neural Mechanisms Involved in Hypersensitive Hearing: Helping Children with ASD Who Are Overly Sensitive to Sounds

**DOI:** 10.1155/2015/369035

**Published:** 2015-12-28

**Authors:** Jay R. Lucker, Alex Doman

**Affiliations:** ^1^Howard University, Washington, DC, USA; ^2^Advanced Brain Technologies, Ogden, UT, USA

## Abstract

Professionals working with children diagnosed with autism spectrum disorder (ASD) may find that these children are overly sensitive to sounds. These professionals are often concerned as to why children may have auditory hypersensitivities. This review article discusses the neural mechanisms identified underlying hypersensitive hearing in people. The authors focus on brain research to support the idea of the nonclassical auditory pathways being involved in connecting the auditory system with the emotional system of the brain. The authors also discuss brain mechanisms felt to be involved in auditory hypersensitivity. The authors conclude with a discussion of some treatments for hypersensitive hearing. These treatments include desensitization training and the use of listening therapies such as The Listening Program.

## 1. Introduction

Professionals working with children who overly react to sounds want to know why the children have hypersensitive hearing. Additionally, parents of these children want to have a better understanding of their children's problems. Often these children are described as being afraid of such sounds, losing control of their behaviors when hearing such sounds, and even fearing that the sounds may be heard when there are no such sounds in the listening environment. Typically these behaviors are seen in children diagnosed with autism spectrum disorder (ASD).

For many people, the method applied to deal with children who have hypersensitive hearing is to avoid the listening situations or deal with the child at the time that the sounds are occurring. Sometimes, people try to prepare children by saying that such annoying sounds will occur; yet, even with this preparation, many of these children still lose control of their behaviors when the actual sounds are heard. Many professionals relate these hypersensitivities in hearing to problems with the children's auditory systems.

Lucker and Doman [1] and Lucker [2] discussed the fact that auditory hypersensitivity in children with ASD is more of an emotional based problem than a specific auditory system problem. In these publications, the authors state that underlying auditory hypersensitivity (or oversensitivity to sounds) involves what are called the nonclassical auditory pathways and limbic system connections. However, the authors do not go very far in describing the brain (or neural) mechanisms involved other than identifying that there is a connection between the auditory system and the limbic system deep in the temporal lobe of the brain. The present paper is a follow-up to these 2012 and 2013 publications. The present paper reviews that what our present research has indicated might be underlying neural mechanisms involved in hypersensitive hearing. Understanding these neural mechanisms will provide the reader with two important factors. First, understanding what is going on with a child who has auditory hypersensitivities is a normal neural reaction and, second, identifying what treatments may be appropriate to help children who are overly sensitive deal more successfully with sound so that they do not have over reactions during listening tasks and, possibly, “shut down” so as to avoid listening and responding to any sounds.

Before beginning the discussion of these various neural pathways, the reader should have a clearer understanding of some of the terms used in this paper. One term is auditory hypersensitivity. This problem is often identified as a person being overly sensitive to sounds. Some professionals have referred to the oversensitivity to sounds as misophonia. Others [3, 4] have called this phonophobia or fear of sound. Thus, in the present paper, the use of hypersensitivity will include oversensitivity to sounds that includes misophonia and phonophobia. It is hypothesized that the same neural mechanisms are involved in all these oversensitivities to sound; however, for some children the sensitivity is only to sounds of an intensity above typical tolerance by people (e.g., greater than 90 dBHL, as 90 dBHL is often identified as a level of maximum tolerance in people). For other children, their oversensitivity may be for a variety of sounds, while for some children it is for specific sounds.

## 2. The Classical Auditory Pathway

**Figure 1:**
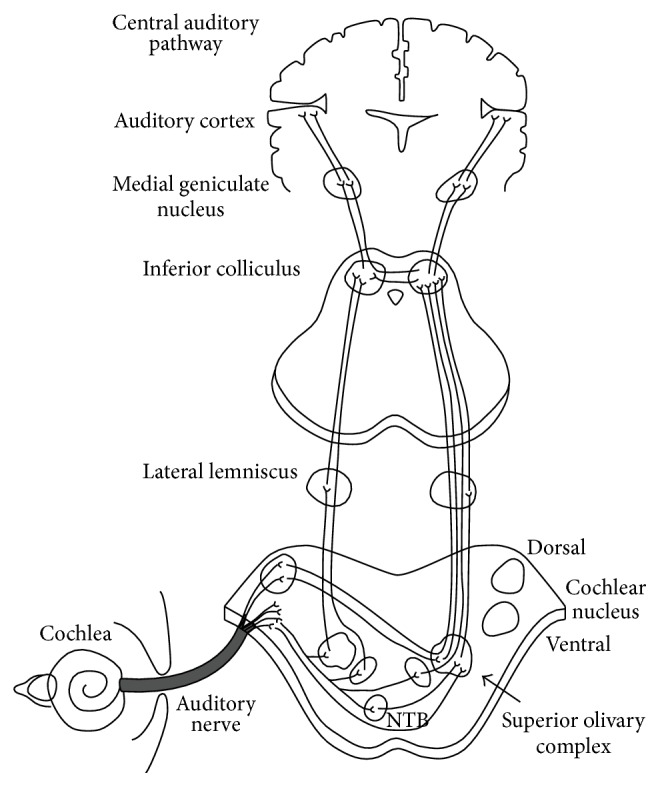
Diagram of the classical auditory pathways from the ear to the auditory cortex (diagram retrieved from https://dnbhelp.wordpress.com/otology/).

Two other important terms used in this paper are classical and nonclassical auditory pathways. Professionals studying hearing typically learn about the standard or classical auditory pathways. These pathways (see Figure 1) start at the cochlea of the inner ear where the cells of hearing, called hair cells, release neurochemicals which are picked up by the auditory nerve also known as the eighth cranial nerve. This nerve can be found in both ears (right and left sides). The auditory nerve travels transmitting the auditory stimulation to a region on the right and left sides of the brainstem called the pons. Specifically, the nerve innervates areas of the cochlear nucleus. Some of the nerve pathways come from the cochlear nucleus project to the same (ipsilateral) side of the brainstem while others cross to the opposite (contralateral) side.

The next level in the system involves a complex of neuronal sites called the superior olivary complex. This is still in the lower brainstem within the pons. From the superior olivary region the nerves travel to the next level called the lateral lemniscus. (This is an important region when we look at the nonclassical pathways.) From the lateral lemniscus, the nerves travel to the inferior colliculus in the classical pathways. The inferior colliculus is at the upper brainstem level, and projections from this level go to the thalamus at the cortical level. The nerves projecting from the medial geniculate bodies project to the auditory cortex which is in the temporal lobe of the brain. Then, via a “bridge” connection between the two auditory cortices of the right and left hemispheres of the brain, the corpus callosum connects these hemispheres so that we can integrate auditory information from each hemisphere.

## 3. The Nonclassical Auditory Pathway

**Figure 2:**
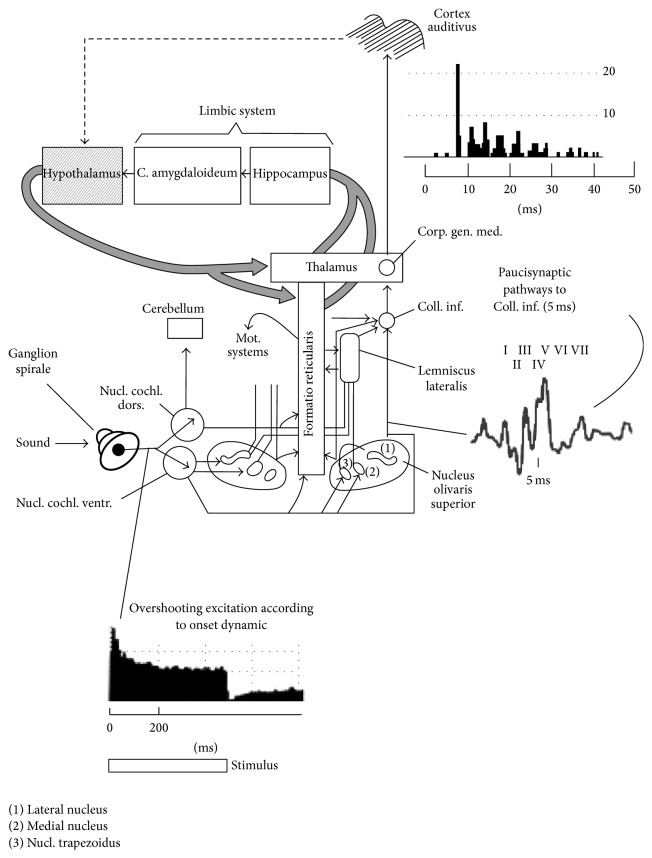
Diagram of the nonclassical auditory pathways showing the relationship between the pathways from the auditory system to the limbic system and to the auditory cortex (diagram retrieved from Noise and Health, 2000, http://www.noiseandhealth.org/viewimage.asp?img=NoiseHealth_2000_2_7_49_31742_1.jpg).

The previous description was of the classical auditory pathways. However, by eight years of age, Moller and Rollins [5] identified that this classical pathway becomes the dominant pathway by which sound travels so that we can process and comprehend what we hear. Moller and his associates [5, 6] identified another pathway which was first investigated in the 1970s which is also an auditory pathway and is mostly active in young children. This pathway has been referred to as the nonclassical auditory pathway (see Figure 2). In typically developing children, by the age of 8 years this pathway becomes suppressed under most listening conditions.

One of the first neural mechanisms identified as being involved in auditory hypersensitivity is this nonclassical pathway. The nonclassical pathway branches off from the classical pathway at the level of the lateral lemniscus. Nerves traveling from the external nucleus of the lateral lemniscus project and receive stimulation from the reticular formation in the upper brainstem. The reticular formation is involved in awareness, detection, and attention to sensory stimulation including sound. The reticular formation then sends neural information back to this region of the lateral lemniscus involved with auditory sensory processing. These neural inputs travel from the external nucleus of the lateral lemniscus to regions of the thalamus but not the medial geniculate bodies which are involved with the classical auditory pathway. From these regions of thalamus, not involved with auditory sensory processing, the nerves project to what is called the auditory association cortex, not the primary auditory cortex which is involved with the major processing of what we hear. These association areas include projections to the limbic system deep within the temporal lobe of the brain. The limbic system is involved with a number of factors including emotional responsiveness. One major center is the amygdala which is involved with emotional memories. What is also interesting is that projections to the thalamic regions that receive input from the external nucleus of the lateral lemniscus go directly to the amygdala. Thus, this nonclassical auditory pathway connects auditory sensory input with the emotional centers of the brain and with brainstem levels of awareness and attention to sensory input.

An important finding from the work of Moller and Rollins [5] and Møller et al. [6] is that not only does the nonclassical pathway obtain auditory sensory information but also it receives stimulation from the somatosensory systems of the body. These systems are involved in processing touch and temperature and help process body position in space. In Moller and Rollins' research [5], they found that overstimulation of touch (sensory processing) in young, typically developing children led to these children becoming overly (hyper) sensitive to sound. This occurred for young children up to 8 years of age. After that age, overstimulation of touch did not lead to an increase in auditory hypersensitivity. However, these were typically developing children. In another study, Møller et al. [6] discussed that children with autism spectrum disorder (ASD) may continue to be overly sensitive to touch and, thus, to sound because their nonclassical auditory systems do not become inhibited to respond under normal listening conditions. In an article published by Musiek et al. [7], they write, “Some children with developmental disorders may have emotional learning problems caused by the brain's inability to reduce the involvement of the non-classical pathway and the amygdala” (p. 6). These children may include those with ASD. These findings support the original discussions from Lucker and Doman's [1] and Lucker's [2] publications that auditory hypersensitivity is largely an emotional (limbic system) based response to sound and not an auditory system response to sound. Thus, the treatment for auditory hypersensitivity, based on this factor, would focus upon calming the emotional system during listening tasks.

## 4. From the Limbic System to Behavioral Reactions to Sound

### 4.1. Involvement of the Vagus Nerve

Once sound has been processed by the brain, the brain reacts to the sound. As noted from the previous discussions, one of the systems that is involved in hypersensitive hearing is the limbic system. Additionally, observing children who show negative behavioral reactions to sounds we see that they tend to react in manners one can call negative emotional behavioral reactions. These negative emotional behavioral reactions can include what we call avoidance or “flight” responses (running away, avoiding, or hiding) or striking out against the “world” or a “fight” response. This “flight and fight” responding is automatic behavior controlled largely by limbic system reactions to stimuli. However, a question arises as to what mechanisms may be involved in this negative behavioral reactivity. One of these mechanisms is through the innervation of an efferent nerve called the vagus nerve or the tenth cranial nerve which receives stimulation from the limbic system.

Porges [8] and Porges and Lewis [9] discuss the functioning of the vagus nerve in setting up automatic bodily responses to sound as part of what Porges calls the “Polyvagal Theory.” This theory relates automatic or autonomic functions in response to incoming stimuli, such as sound. These autonomic functions are regulated through what is called the autonomic nervous system or ANS. Essentially, this theory describes how the vagus nerve is innervated through limbic system connections. The information transmitted via the vagus nerve innervates bodily responses to various emotional states including stress and fear. For example, the vagus nerve innervates the heart and, under stress and fearful conditions, a person's heart rate increases. The vagus nerve also innervates the larynx or vocal folds which allow people to vocalize and speak. Under stress and fearful conditions, one may notice how a person's voice “cracks,” raises in pitch, or becomes weak. When sound causes people to have negative emotional reactions (i.e., hypersensitivity), the vagus nerve may become innervated by the limbic system leading to ANS reactions of “flight and fight” which are often seen in children who overly react to sounds, such as children with ASD. Also, these children may “shut down” in listening and start babbling incoherently. These changes may be affected by vagal nerve innervation.

### 4.2. Other Reactions Based on Limbic System Innervation

Mazurek et al. [10, 11] discussed their research looking at the effects of stress (increased negative emotionality) in rats. They found that under stress, these rats were highly oversensitive to sound (i.e., auditory hypersensitivity). They identified that there were negative reactions after hearing these sounds in these “stressed” rats. The reactions were measured in their hypothalamus (a brain mechanism part of the limbic system), their pituitary glands, and their adrenal glands (the latter two being part of the ANS). This research suggests that children who are hypersensitive to sound may have problems because of similar reactions in their hypothalamus, as well as their amygdala; both areas are identified earlier in this paper as part of the limbic system. Furthermore, the reactions to these nontolerated sounds can set off ANS responses as well as the vagal nerve reactions previously discussed.

In the initial study by Mazurek et al. [10] discussed above, the stress in their lab rats was produced by exposure to annoying sounds that were not very intense. This could be related to child's normal, ANS reactions to sounds which are not tolerated which lead to infants and very young children being frightened by these sounds. For children with ASD, their limbic systems may develop negative memories of these sounds (through the amygdala) and react negatively because of ANS innervation as seen with the animal experiments discussed.

## 5. Other Neural Mechanisms Involved with Hypersensitive Hearing

The nonclassical auditory pathways and the limbic system are important neural mechanisms involved in hypersensitive hearing in children, especially those with ASD. However, there has been recent research indicating that some other factors may be involved. One of these other factors involves problems with neural innervation due to abnormal production of neurochemicals such as GABA or gamma-aminobutyric acid as well as 5-hydroxytryptamine (5-HT). This problem with neurochemical production may further increase the auditory hypersensitivities in children. What the research indicates is that this abnormal production is found in children with ASD.

### 5.1. Abnormal Neurochemical Production and Inhibition

#### 5.1.1. GABA

Rubenstein and Merzenich [12] identified that one of the neural mechanisms found in people with auditory hypersensitivity is a problem with the neurochemical GABA. They discuss how abnormal GABA production has been found in children with autism. Furthermore, they explain how GABA helps in the inhibitory control of sensory processing including auditory sensory processing.

Inhibition relates to functioning in the following manner. When we receive incoming sensory stimuli, we can allow the stimulation to be transmitted from the sensory receptive organ (such as the inner ear) to the brain for further processing. In the brain, there are cells that transmit neurochemicals that “block” the transmission of the incoming sensory stimulation. This blocking is called inhibition which leads to a reduction in responding. Thus, in the case of nontolerated, annoying sounds, the brain can “block” or reduce the transmission of the incoming auditory sensory stimuli from getting to the receptor centers in the brain. One neurochemical that does this blocking is GABA. Rubenstein and Merzenich [12], as well as investigations by others cited by these authors, have identified that release of GABA in the brain inhibits overreaction to sensory stimulation such as sound. Furthermore, they discuss that children with ASD have a reduction in their GABA production. Abnormal GABA production and transmission leads to a hyperreactivity in the neurons of the brain because of this lack of inhibitory responding. Thus, the person's brain is not appropriately processing sounds, and the body's reaction to this may be referred to as overreactivity. This overreactivity can lead to negative emotional reactions that lead the child with ASD to fear and dislike sounds they hear. Research is needed to investigate GABA in children with autism and identify whether hypersensitive hearing may be due to abnormal production of GABA, inappropriate transmission of this neurochemical, or a combination of both factors.

#### 5.1.2. 5-Hydroxytryptamine (5-HT), Serotonin

Marriage and Barnes [13] identified that another neurochemical involved in sensory modulation and, thus, sensory processing, is 5-HT, also known as serotonin. Based on animal studies, these researchers describe that abnormal functioning of 5-HT caused a heightened startle response to auditory stimuli in animals. This suggested that auditory hypersensitivity in people might be due to abnormal functioning of 5-HT (serotonin). Baguley [14] stated that people with William syndrome, who are typically found to have auditory hypersensitivities, also have serotonin abnormalities in their brains. As such, it is possible that children with ASD may also have abnormal production of 5-HT. Further research is needed to look at 5-HT/serotonin functioning in these children and relate this to listening and auditory hypersensitivities.

### 5.2. Sensory Gating

When we consider the neurochemical factors contributing to inhibition, we can see that these factors may relate to what some people call sensory gating. Some of the behavioral reactions we see in children who are hypersensitive to sound may involve how the brain “gates” or deals with sensory information such as sound and, due to this gating, how the brain sets up behavioral reactions to sound. Gating relates to the regulation of how the brain handles incoming sensory information. This “gating” deals with two factors related to sound processing. One is the acceptance and “passing on” of sound information to higher cortical centers such as when we listen in conversations, our neural sensory gating system allows what the speaker is saying to be passed through the auditory system to higher levels of auditory processing into the language centers for language processing and on to the cognitive centers where we think about what the speaker is saying. At the same time, there may be background noises which our sensory gating systems inhibit so they are not processed beyond a certain low neural level. This is often referred to as auditory filtering.

Some children with auditory filtering problems are described as having abnormalities in something called executive functioning or self-regulation. Executive functions involve those regulatory mechanisms in the prefrontal cortex that send neural messages to other parts of the brain to manage the processing of information, including what we hear. These abnormalities are deficits in the frontal lobe of the brain which suggest that children with ASD having auditory hypersensitivities may have abnormalities in this part of their brains.

Orekhova et al. [15] and Mayer et al. [16] discuss this sensory monitoring or gating and state that our reactions to how we respond to sensory input are strongly related by prefrontal cortical activity. Abnormal prefrontal cortical functioning can contribute to poor sensory gating and overreacting to sensory stimuli. Thus, one factor in the frontal lobe contributing to why children may react so strongly to sound (which we call auditory hypersensitivity) is poor executive functioning which does not allow their brains to inhibit these negative emotional reactions from occurring.

### 5.3. Abnormal Functioning in the Frontal Lobe of the Brain

Another group of researchers have investigated what may be involved with frontal lobe malfunctioning. Gomot et al. [17] looked at the electrophysiological measure called the mismatched negativity or the MMN response which is related to functioning in the frontal lobe of the brain and is a response that occurs just prior to neural activity related to listening attention. The MMN response is a discriminatory response to changes in auditory stimuli. For example, if two tones of the same frequency or two monosyllabic words are heard, there is a relatively low neural response measured by the MMN. Then, if one of the tones or words is changed, the MMN becomes larger indicating that the brain has discriminated and identifying the difference.

Gomot et al. [17] found that in children with auditory hypersensitivities, the MMN from the left frontal lobe was abnormal compared with children who had normal reactions to high intensity sounds. Thus, one can conclude that children with auditory hypersensitivities may not be able to appropriately discriminate changes in loudness in sound so that unexpected changes may lead to negative emotional reactions. Thus, one other factor that may lead to abnormalities in brain (neural) functioning in children with ASD is this abnormal functioning in discriminating sound. Further research is needed looking at the MMN in children with ASD.

## 6. What May Occur in Children with Auditory Hypersensitivity

Children's reactions to certain, intolerant sounds may be a natural, negative response which, over time, becomes filtered by the brain mechanisms discussed above. When we think about how parents (and others) react to young children's responses to sounds the child cannot tolerate, the reactions are usually hugging the child, comforting the child through touch, and similar physical reactions. As discussed earlier, somatosensory information is integrated with sound in the nonclassical auditory system. In the typically developing child, these manual touches may be processed as comforting leading to a reduction in stress which then becomes processed through the nonclassical auditory system to interpret sounds that are annoying and intolerant as not being fearful. Thus, the child learns not to fear such sounds and not to be stressed out when hearing or expecting these annoying sounds. In contrast, it is hypothesized that the child with ASD and auditory hypersensitivities may overreact not only to the sounds that cause the negative behavioral reactions but also to somatosensory stimulation, so that when people try to comfort them as young children, their nonclassical auditory systems react with further negative responses, and they learn to associate negative emotional reactions to the sounds. Thus, a focus of the treatment for auditory hypersensitivity has to be to reduce this negative emotional reactivity, reduce the stress, and reduce the “fight or flight” reactions in people with hypersensitive hearing.

## 7. Treatment for Hypersensitive Hearing

In view of the above discussions, the underlying theme is that hypersensitive hearing involves neural mechanisms that are related to emotional processing, emotional memory, and emotional reactivity which is considered negative. This negative reactivity can lead to fight/flight responses affecting the autonomic nervous system and secretion by glands throughout the body as well as neural motor responses that can lead to a deterioration or loss of vocalizing (vagus nerve reactions) as well as behavioral responses of fighting and running away. As such, the underlying factor for treating these problems is to focus on the negative emotional reactions.

In order to better understand how one focuses on the emotional responses, one needs to have a basic understanding of behavioral therapies. There are two general approaches to behavioral therapy. One is called operant conditioning and the other is called classical conditioning. Operant conditioning is typically associated with the work of BF Skinner while classical conditioning is associated with the work of Ivan Pavlov. Operant conditioning follows the basic concept that when we are presented with a stimulus (e.g., a sound of high intensity) and there is a response (e.g., the child running away), there is a reaction which occurs that reinforces or punishes the response increasing (reinforcement) or decreasing (punishment) the likelihood that the response will occur again when the same stimulus is presented. The therapist, educator, or parent providing the reinforcement presents a reaction to the child's behavior in order to reduce the running away behavior (punishment) or to reinforce the child listening to the sound (reinforcement). Classical conditioning works slightly differently. We present a stimulus (e.g., the same intense sound) but we teach the child a behavior that is different from what is called the unconditioned response (in this example, the child running away is the unconditioned response). We may teach the child to count how long the sound is present and reinforce this conditioned response. Over time, the expectation is that the child hearing the sound will just count how long it lasts, but not run away. When we consider retraining or reprogramming children who have auditory hypersensitivity, we need to think more in line with classical conditioning for children who are responsive and can handle such behavioral training. For some children, such as those with ASD, we may need to consider operant conditioning treatments similar in many respects to what is often used with these children called ABA or applied behavioral analysis.

Therapy or training, which can also be called retraining, is to associate the negative sounds with some behavior that will lead to a more calming response and, thus, reduce the negative emotional reactions to the sound. There are two proposed methods for doing this. One is through a straight behavioral training program. The other is through the use of a listening therapy. The following is a discussion of each of these approaches.

### 7.1. Behavioral Training for Sound Desensitization

The underlying concept of a behavioral approach is to recondition the person's system so that the emotional memory is changed from negative to more neutral and, if possible, positive. Then, if the person is faced with those stimuli which originally “set off” the negative emotional responses, the person would have a more neutral response. The goal of behavioral training is to desensitize the person to the previously feared sounds. An example of a behavioral approach would be as follows. Consider a person being annoyed by sounds and having strong negative emotional reactions to people eating because of the sound of crunching food. The person might be taught how to relax his/her body and feel more calm which would lead to a more positive emotional feeling. Then, the person would engage in the relaxation exercise while hearing food crunching first presented at a low volume level for short periods of time then having the volume and length of time increased. Eventually, the person would start associating this relaxed, more positive feeling with the crunching sound and feel that he/she can control his/her reactions to the sound.

As one can see, this desensitization using a behavioral approach takes time and a level of involvement on the part of the person that may not be available for many children with ASD. For them, another approach might be more successful. This approach may be called “in vivo exposure.” In this approach, the person who presents with hypersensitive hearing is slowly introduced to the sounds and situations involving the sounds that led to the negative emotional reactions. This is done in a controlled manner in real life (in vivo) situations. For the same example as used above, the person may be exposed to someone softly crunching food once. The volume of crunching and the number of crunches as well as the length of time having to listen would then be systematically increased until the person is able to sit through a meal with people. The child going through this systematic desensitization training would be rewarded for success in sitting still while the person is hearing the food crunching sound (an operant conditioning approach).

An example of in vivo exposure is seen in the research of Koegel et al. [18]. In their research, they used this approach with a small group of children identified as being on the autism spectrum and found that even one year after training, the children were desensitized to the sounds which they originally feared. Furthermore, generalization of the training occurred so that the children became desensitized to many sounds that were not part of the original training program.

### 7.2. Use of Listening Therapies

Along with desensitization training (whether a classical conditioning approach or in vivo exposure is used) one can use a sound intervention or listening therapy. A listening therapy is the use of specially recorded music and environmental sounds having nothing necessarily to do with the sounds to which the person has negative emotional reactions. The music and recorded sounds are carefully chosen and systematically produced to enhance positive emotional and calming reactions when listening. This type of training involves a more passive method to desensitize the limbic system and reprogram the emotional memory system with the aim of making sounds something to which one desires to listen rather than avoid. The listening method described here uses specially recorded instrumental music. The music is acoustically modified to lead the child to react less negatively to sounds and, thus, reduce the child's hypersensitivity.

Although a number of listening methods have been developed, one specific method found beneficial for children with ASD is The Listening Program (TLP). In TLP, a trained provider, usually a professional such as an occupational therapist or speech-language pathologist, oversees the training. The provider typically establishes the actual protocol based on the individual child's needs and may carry out the training in the child's home or at the child's school. When using TLP, especially through specially designed air and bone conduction headphones, the sound signal is believed to travel along both classical and nonclassical auditory pathways. One of the first outcomes that parents, educators, and professionals often see in children undergoing TLP training is that the children are calmer. This is a good indicator that the listening has tapped into the emotional areas of the limbic system via the nonclassical auditory pathways. Over the course of training, children often are reported to be more attentive to sounds, better able to detect (discriminate) sounds they hear, and are more verbally communicative likely because they are more open to listening. As the training proceeds, the child continues to relax and become calmer when listening. It is hypothesized that this is because a reprogramming of emotional memory in the amygdala is occurring. The training also helps improve stress regulation so the child no longer has fight/flight responses to nonthreatening sounds. When the child then finds him- or herself in real-world situations and hears sounds that may have been frightening or annoying in the past, the training allows the child to process the sounds in a more neutral manner.

For many children, the use of a program such as TLP is sufficient to reprogram their systems so that sound is no longer frightening. The child may still react verbally saying that he or she does not like such sounds or the situations in which the sounds occur, but after TLP training, the child should no longer lose control of his or her behavior when hearing such sounds. Additionally, the child may be more willing to listen to parents or teachers who can model desensitization by saying, “The sound is annoying, so let's move over here,” or “If we walk more quickly toward [our goal], we can get away from having to hear that sound.”

Research using TLP has largely been anecdotal, but the evidence base is growing as studies continue to be conducted. For example, Gee et al. [19] present a case study of a child with ASD who was overly sensitive to sound and completed TLP training. They report that the child showed a decrease in negative behavioral reactions to previously “annoying” and feared sounds after completing this training. A review of other studies and descriptions of individual cases can also be found at the Advanced Brain Technologies website (http://www.advancedbrain.com/). The research has demonstrated that after going through this listening program, children, adolescents, and adults find their abilities to tolerate sounds are improved. Many find listening to be more pleasurable and positive so that negative emotional reactions rarely occur. Thus, professionals working with children who have ASD and auditory hypersensitivities as well as parents of these children might wish to try TLP training on an investigative basis focusing on seeing changes in the children's reactions to sounds in their environment. It is the expectation of these authors that completing TLP training should lead to reduced negative emotional reactions when listening increasing the child's listening and communication skills. Since listening is an important part of learning, it is hoped that the improvements in listening will improve learning for these children as well. Further research is needed carefully investigating these educational and communicative factors.

## 8. Conclusions

The underlying theme throughout this paper is that the primary neural mechanisms in hypersensitive hearing involve negative emotional reactions to sound via connections between the auditory system and the limbic system as well as the limbic system and other parts of the body. Research has demonstrated issues involving the auditory system connecting with the limbic system as well as frontal lobe involvement that can contribute to negative emotional reactions. Research has also identified some problems based on poor neurochemical functioning, specifically GABA and serotonin, which eventually lead to negative emotional reactions when listening.

Children with ASD may have negative emotional reactions to some sounds (i.e., auditory hypersensitivity) that can involve some degree of a fight or flight responses. The treatment for these hypersensitive hearing problems is felt to focus on changing the negative emotional reactions to more neutral and, hopefully, positive responses. This can be done through desensitization training which can be in an active form of behavioral reconditioning or a holistic form called in vivo exposure. Additionally, the use of a listening therapy, such as The Listening Program, can lead to more neutral or positive reactions when listening to these annoying sounds.

The focus of this paper was to help the reader understand the underlying mechanisms involved in hypersensitive hearing and the treatments that can help reprogram the person's mechanisms to be more open to listening. In the end, the focus is to help the child with ASD be more involved in communicating with the world around him/her. It is hoped that professionals and parents will consider these factors and the treatment recommendations discussed. Future research should focus on these factors and their direct relationship to hypersensitive hearing in children with ASD so that we can obtain a greater understanding of specific mechanisms involved with hypersensitive hearing and the evidence base for the use of treatments such as desensitization training and listening therapy.
